# Deep Learning Method for Precise Landmark Identification and Structural Assessment of Whole-Spine Radiographs

**DOI:** 10.3390/bioengineering11050481

**Published:** 2024-05-11

**Authors:** Sung Hyun Noh, Gaeun Lee, Hyun-Jin Bae, Ju Yeon Han, Su Jeong Son, Deok Kim, Jeong Yeon Park, Seung Kyeong Choi, Pyung Goo Cho, Sang Hyun Kim, Woon Tak Yuh, Su Hun Lee, Bumsoo Park, Kwang-Ryeol Kim, Kyoung-Tae Kim, Yoon Ha

**Affiliations:** 1 Department of Neurosurgery, Ajou University College of Medicine, Suwon 16499, Republic of Korea; 2 Department of Neurosurgery, Yonsei University College of Medicine, Seoul 03722, Republic of Korea; 3 Promedius Inc., Seoul 05609, Republic of Korea; 4 Department of Neurosurgery, Hallym University Dongtan Sacred Heart Hospital, Hwaseong-si 18450, Republic of Korea; 5 Department of Neurosurgery, Pusan National University Yangsan Hospital, Busan 50612, Republic of Korea; 6 Department of Neurosurgery, Bon Hospital, Daejeon 34188, Republic of Korea; 7 Department of Neurosurgery, Daegu Catholic University College of Medicine, Daegu 42400, Republic of Korea; 8 Department. of Neurosurgery, School of Medicine, Kyungpook National University, Kyungpook National University Hospital, Daegu 41944, Republic of Korea; 9 Department of Neurosurgery, Spine and Spinal Cord Institute, Severance Hospital, Yonsei University College of Medicine, Seoul 03722, Republic of Korea

**Keywords:** artificial intelligence, deep learning, radiography, spine

## Abstract

This study measured parameters automatically by marking the point for measuring each parameter on whole-spine radiographs. Between January 2020 and December 2021, 1017 sequential lateral whole-spine radiographs were retrospectively obtained. Of these, 819 and 198 were used for training and testing the performance of the landmark detection model, respectively. To objectively evaluate the program’s performance, 690 whole-spine radiographs from four other institutions were used for external validation. The combined dataset comprised radiographs from 857 female and 850 male patients (average age 42.2 ± 27.3 years; range 20–85 years). The landmark localizer showed the highest accuracy in identifying cervical landmarks (median error 1.5–2.4 mm), followed by lumbosacral landmarks (median error 2.1–3.0 mm). However, thoracic landmarks displayed larger localization errors (median 2.4–4.3 mm), indicating slightly reduced precision compared with the cervical and lumbosacral regions. The agreement between the deep learning model and two experts was good to excellent, with intraclass correlation coefficient values >0.88. The deep learning model also performed well on the external validation set. There were no statistical differences between datasets in all parameters, suggesting that the performance of the artificial intelligence model created was excellent. The proposed automatic alignment analysis system identified anatomical landmarks and positions of the spine with high precision and generated various radiograph imaging parameters that had a good correlation with manual measurements.

## 1. Introduction

Recently, there has been a remarkable surge in the availability of biomedical data, presenting challenges and opportunities for healthcare research. This wealth of data includes extensive collections of medical images, such as computed tomography (CT) scans, magnetic resonance imaging (MRI), and radiographs, which play a crucial role in various medical tasks, such as pathology detection and classification, as well as pinpointing vital anatomical landmarks. Spine imaging, in particular, holds significant clinical importance as it enables the precise characterization of spinal alignment through angles, distances, and shapes, proving invaluable for tasks such as surgical planning and monitoring of deformity progression [1]. Traditionally, these parameters are measured either manually using tools such as rulers and protractors on physical images or with specialized software for digital images [2]. However, this approach is prone to inaccuracies and inconsistencies due to variations in measurements by different observers.

To address these challenges, there has been a growing emphasis on developing computer-aided diagnosis systems over the past few years. These systems aim to reduce errors and enhance the efficiency of image analysis; however, they often require manual input [3]. The advent of fully automated software tools promises to eliminate these shortcomings and revolutionize both medical research and clinical practice. Recent advancements in deep learning (DL) technologies, coupled with the high computational capabilities of graphics processing units (GPUs), have made it feasible to develop tools capable of autonomously measuring spinal parameters [4,5]. These technological advances not only streamline the analysis process but also enhance its accuracy, paving the way for more precise and reliable medical diagnostics and treatments.

In medical imaging, the integration of artificial intelligence, particularly DL, has significantly increased in recent times, often surpassing the expertise of human observers in terms of performance. One notable advancement was the development of an automatic tool for identifying vertebrae in CT scans [6]. This tool accurately pinpointed vertebral centroids but fell short of providing practical clinical applications. In a study by Jacobsen et al., DL was employed for the automatic segmentation of cervical vertebrae [7]. However, their methodology exhibited non-negligible errors in locating the vertebral corners, and the focus was limited to the cervical area with a relatively small dataset, hindering its practicality in routine clinical environments.

To address the limitations of these studies, we aimed to develop an artificial intelligence model to accurately identify points from which to perform key measurements on whole-spine radiographs. This study aimed to measure each parameter automatically by accurately marking the point for measuring each parameter on whole-spine radiographs.

## 2. Materials

### 2.1. Dataset

Between January 2020 and December 2021, a comprehensive collection of 1017 sequential lateral whole-spine radiographs was retrospectively gathered. In adherence to the guidelines of our hospital’s institutional review board (IRB no. 2023218), a waiver for informed consent was granted for this study. A leading radiologist meticulously reviewed the entire set of images and excluded several categories: (1) insufficient length, failing to capture either the C2 dens or both femoral heads; (2) anatomical variances, such as spinal columns with less or more than the standard 25 vertebrae; and (3) compromise by suboptimal contrast, hindering clear identification of pelvic structures.

**Figure 1:**
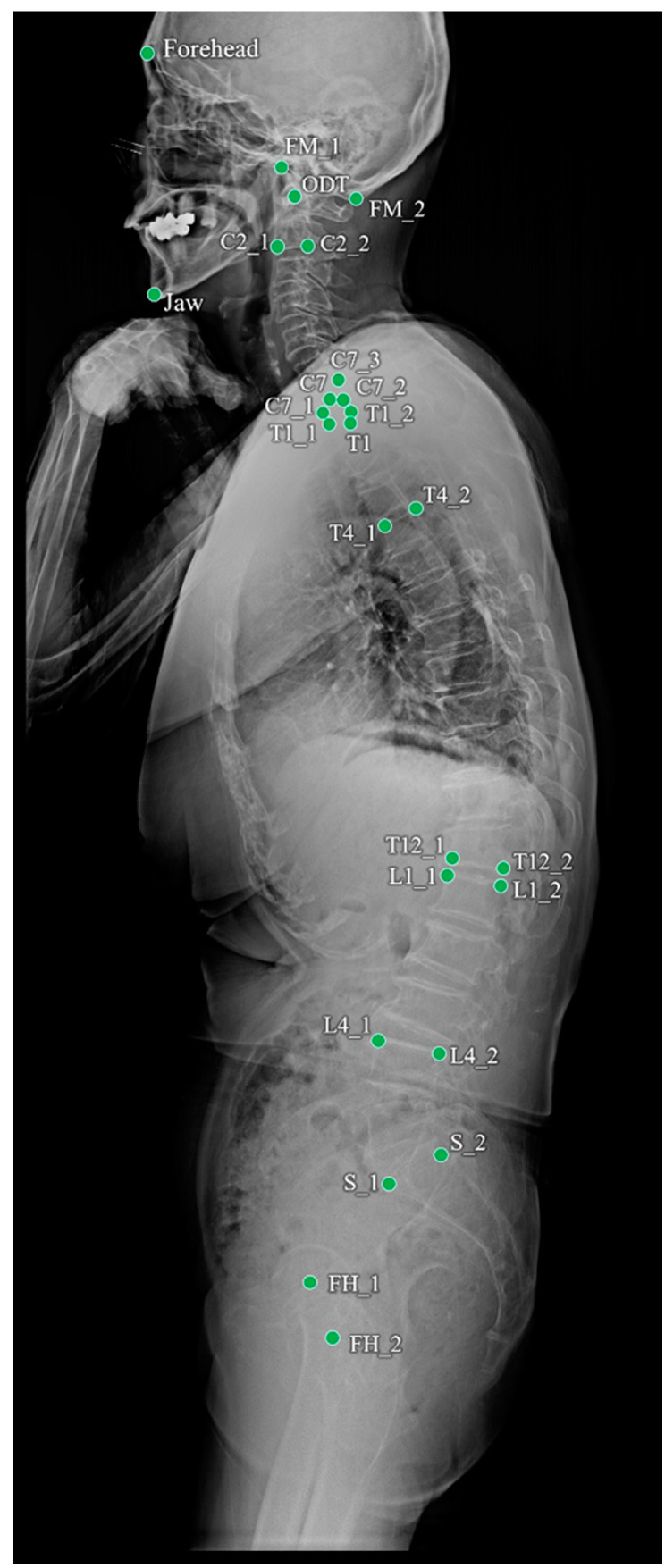
Landmarks annotated on a whole-spine lateral radiograph.

Of the 1017 radiographs, data from 819 and 198 were used for training and testing the performance of the landmark detection model, respectively. To objectively evaluate the performance of the program, 690 whole-spine radiographs from four other institutions were used for external validation. The annotated landmarks contained 26 points, as shown in Table 1 and Figure 1. The demographic profile for these 1707 annotated images revealed a mean patient age of 42.2 ± 27.3 years (age range: 20–85 years) at the time of the radiographic examinations.

### 2.2. Learning of Heatmap-Based Landmark Detection

**Figure 2:**
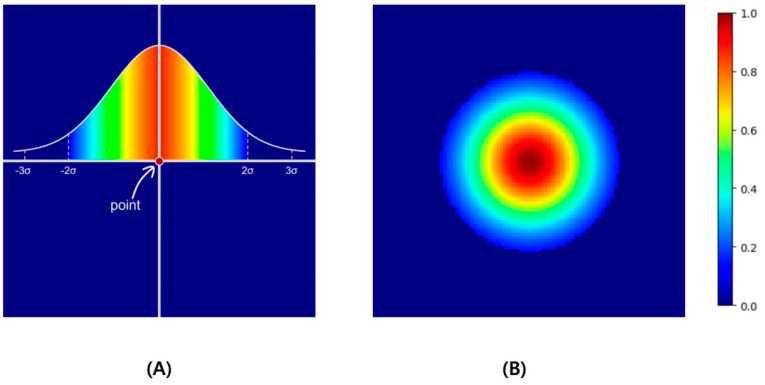
As an example, if the point located in the center of 100 × 100 as in (**A**) is expanded to Gaussian values and normalized to values 0 to 1, a heatmap like (**B**) is created. In this example, σ was set to 10, thresholded in the ±2σ range, and visualized with a jet colormap.

The model for detecting landmarks used U-Net [8], and learning was conducted based on a heatmap. The heatmap-based method indirectly learns coordinates through heatmaps instead of directly learning them. This method is widely used in landmark detection for pose estimation [9] or face landmark detection [10]. Heatmap-based learning is slower than the direct prediction of coordinates, but it is less sensitive to slight differences that may occur owing to human annotations because it accepts the surroundings of coordinates more generously. The proposed heatmap-based landmark detection model used a Gaussian heatmap generated around landmark coordinates as the ground truth (Figure 2), and the dice coefficient loss ($L_{dice}$) and weighted L1 loss ($L_{wl}$) were used as the loss functions [11].

(1) $$L={\alpha L}_{dice}+\beta L_{wl}$$

L1 loss, which is the absolute difference between the ground truth and the prediction, leads to a predicted heatmap ($\hat{Y}$) similar to the ground truth (Y). However, compared with the overall image size, a single point is very small. Therefore, we categorized the area of the point as the foreground and the area outside the point as the background. Subsequently, we applied the weighted L1 loss by assigning weights that were inversely proportional to each foreground and background area. The background (bg) and foreground (fg) were determined based on 2σ of the simulated Gaussian.

(2) $$fg(x)=\left\{\begin{array}{c} 0,\;x\leq 2\sigma \\ 1,\;x>2\sigma \end{array}\right.\;bg(x)=\left\{\begin{array}{c} 1,\;x\leq 2\sigma \\ 0,\;x>2\sigma \end{array}\right.\;L_{wl}=\sum \left(W*\left|\left.Y-\hat{Y}\right|\right.\right)\;W=fg\left(Y\right)/\sum \left(fg\left(Y\right)\right)+bg\left(Y\right)/\sum \left(bg\left(Y\right)\right)$$

Dice loss was added to bring the predicted heat map closer to the ground-truth Gaussian-distributed heat map. This loss is inversely related to the dice similarity coefficient (DSC), which measures the similarity between two samples.

(3) $$y=fg\left(Y\right)*Y\;\hat{y}=fg\left(\hat{Y}\right)*\hat{Y}\;L_{dice}=1-DSC(y,\;\hat{y})$$

DSC has a value between 0 and 1. The higher the similarity, the closer it is to 1, and the lower the similarity, the closer it is to 0. DSC calculates only the foreground area of each sample, and in this case, the foreground is an area divided by 2σ as a boundary, similar to weighted L1 loss.

(4) $$DSC\left(y,\;\hat{y}\right)=\frac{\sum \left[\left(y+\hat{y}\right)*\left(y*\hat{y}>0\right)\right]}{\sum y+\sum \hat{y}}$$

Finally, the landmark coordinate outputs from the model were the center points of the maximum values from the predicted heatmap.

### 2.3. Workflow of the Landmark Detection in Whole-Spine Lateral Radiographs

**Figure 3:**
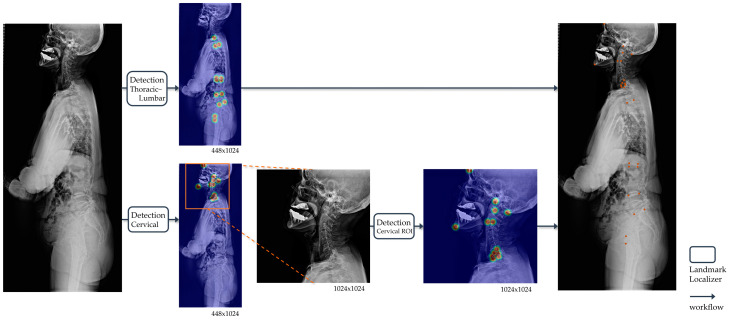
Operational flow of the landmark detection model in whole-spine lateral radiographs. For automatic landmark detection in a single radiograph, the thoracic–lumbar and cervical spine are localized separately. The outputs of landmark localizers for each image input are all heatmaps, and the final output of the model is the coordinates (orange points) restored to match the original image resolution derived from the heatmaps.

The landmark detection model in whole-spine lateral radiographs was divided into two steps: detection of the upper cervical area above T1 and the lower thoracic–femur area (Figure 3). This aimed to achieve precise detection of densely clustered landmarks in the cervical area, which have a higher density than the resolution of the entire image. The detection of the cervical area was further divided into two steps. First, the cervical region of interest (ROI) within a whole-spine radiograph was identified. The cervical ROI range was specified with a margin of 30% of the horizontal margin in a tightly bound box from 13 landmarks above T1 detected on the whole-spine radiograph. In the second step, detection was performed at a higher resolution in the cervical ROI. Finally, the predicted landmarks of the whole-spine radiograph were derived by combining the prediction points of the detection model in the thoracic–femur area and the detection model in the cervical ROI.

### 2.4. Training Details

**Figure 4:**
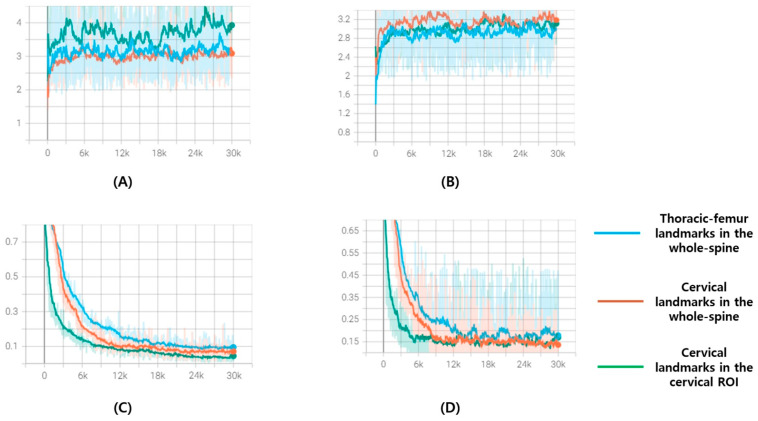
These are loss and accuracy curve graphs for 300 epochs of the training set and validation set: (**A**) Accuracy curve graphs of training set; (**B**) accuracy curve graphs of validation set; (**C**) loss curve graphs of training set; (**D**) loss curve graphs of validation set. The *x*-axis represents steps and is plotted at every 100 steps. Blue is the curve of the model that finds thoracic–femur landmarks in the whole spine, orange is the curve of the model that finds cervical landmarks in the whole spine, and green is the curve of the model that finds cervical landmarks in the cervical ROI.

The input image size was set to 448 × 1024, whereas the cervical ROI training model used a 1024 × 1024 resolution image as the input. All the inputs were resized while maintaining the aspect ratio (height/width), and pixel values were rescaled by referring to the windowing information in the whole spine radiograph DICOM header, after which contrast limited adaptive histogram equalization (CLAHE) was applied. All inputs were resized by maintaining the aspect ratio and then rescaled and inputted after applying CLAHE. Augmentation during training was shift (±10%), zoom (±10%), and rotation (±10°). The sigma (σ) for heatmap generation was set to 10 and 15 for the whole-spine lateral radiograph and cervical ROI, respectively. Dice loss could be applied after a certain amount of training, so the α in the loss function started from 0 and increased by 0.002 per epoch, while β was set as 1 − α. All models were trained in an Ubuntu 22.04.4 LTS, Intel^®^ Core™ i9-9900X CPU @ 3.50GHz x4ea, a single GPU environment [Quadro RTX 8000 (48 GB)], and the TensorFlow 2.11 version was used as a framework. Of the 819 training sets, 794 were used to update the model’s weights, and 25 were used as validation sets during training. After running 300 epochs with a batch size of 10, the weight at the epoch with the lowest average validation loss in the cumulative 10 epochs was selected as the final weight of the detection model. Figure 4 shows the loss and accuracy graph monitored for each 100 steps during the learning process.

### 2.5. Measurement of Spinal Parameters

Fifteen spinal parameters were measured from the landmarks detected in whole-spine lateral radiographs using the landmark detection model. The names and measurement methods for these parameters are listed in Table 2.

### 2.6. Statistical Analysis

The landmark localization errors were used to evaluate the performance of the trained landmark localizer. Interrater reliability was used to determine the level of agreement among the following three raters:

Rater 1 (R1): Senior neurosurgeon

Rater 2 (R2): Junior neurosurgeon

Proposed DL model (landmark localizer and numerical algorithm)

In this study, Pearson correlation coefficients were employed to assess the relationships between the predicted radiographic parameters using a DL model and the actual ground truth values. To determine the numerical discrepancies between the model predictions and ground truth, Wilcoxon signed-rank tests were utilized, with a *p*-value threshold of <0.05 indicating statistical significance. Furthermore, the intraclass correlation coefficient (ICC) was used to measure the interobserver reliability of three human evaluators (junior resident, spine fellow, and senior surgeon), the DL model, and ground truth. This analysis was based on a dataset of 198 images specifically chosen for interobserver reliability evaluation. The reliability was categorized into four levels based on the ICC value: excellent (0.9–1.0), high (0.7–0.9), moderate (0.5–0.7), and low (0.25–0.5). All statistical analyses and procedures in this research were performed using SPSS version 25.0 (SPSS Inc, Chicago, IL, USA)

## 3. Results

### 3.1. Dataset Demographic

The dataset comprised radiographs from 857 female and 850 male patients, with an average age of 42.2 ± 27.3 (range: 20–85) years. In this dataset, spinal implants were present in 170 images (approximately 10%), with the range of instrumentation extending from C4 to the ilium, averaging 8.2 ± 3.0 levels per image.

### 3.2. Performance of the Landmark Localizer

**Figure 5:**
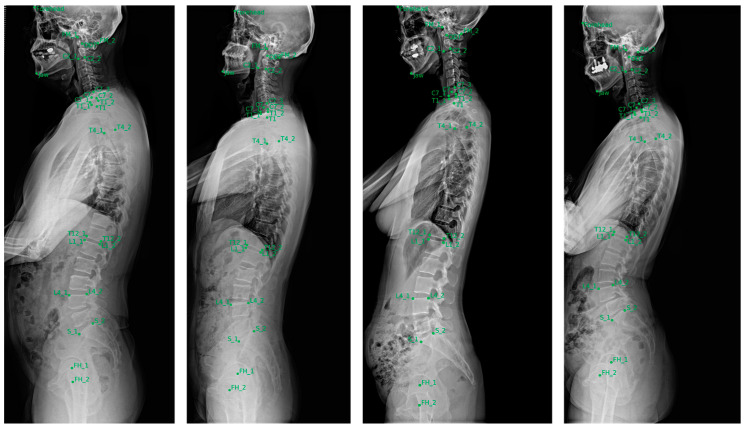
Examples of landmarks automatically localized in the test set.

The landmark localizer showed the highest accuracy in identifying cervical landmarks, with a median error of 1.5–2.4 mm. This was followed by the lumbosacral landmarks, which exhibited a median error of 2.1–3.0 mm. In contrast, the thoracic landmarks displayed larger localization errors, with median values of 2.4–4.3 mm, indicating slightly reduced precision compared with the cervical and lumbosacral regions. Figure 5 shows a visualization of localized landmarks in the test set.

### 3.3. Inter-Rater Reliability between the Two Human Experts and Developed Deep Learning Model

Table 3 shows the inter-rater reliability of the spinal curvature characteristics between the two human experts and the developed DL model. The consistency in measurements between the senior and junior neurosurgeons was outstanding across all spinal curvature characteristics, with all ICCs exceeding 0.9, indicating excellent agreement. When compared with the evaluations made by human experts, the proposed DL model showed slightly lower reliability in accurately predicting the cervicothoracic junction point and the degree of thoracic kyphosis. However, its performance in determining the thoracolumbar junction, cervical and lumbar points, and lumbar lordosis was comparable with that of human experts. Overall, the agreement between the DL model and the two experts ranged from good to excellent, with ICC values exceeding 0.88.

### 3.4. Performance Evaluation of the Spinal Parameters of the Deep Learning Model

The performance of the DL model in estimating spinopelvic parameters was rigorously evaluated using a test dataset comprising 198 spinal radiographic images. The results, outlined in Table 4, show mean errors for these parameters, considering the non-normal distribution of error values. The mean errors were accompanied by the standard deviation.

All predicted radiographic parameters demonstrated significant correlations with the ground truth values, with *p*-values less than 0.001. For core spinopelvic parameters, the mean error varied from 0.16° for odontoid hip axis angle (ODHA) to 5.69° for lumbar lordosis. Notably, no significant differences were found between the model predictions and ground truth values, as evidenced by all *p*-values > 0.05 in the Wilcoxon signed-rank tests. The predicted Chin-Brow Vertical Angle (CBVA) and pelvic incidence (PI) were particularly well correlated with the ground truth, exhibiting Pearson correlation coefficients (R) > 0.9. When examining regional spinal parameters, performance varied across anatomical regions. In the cervicothoracic region, the mean errors spanned from 0.66° for cervical CBVA to 5.66° for T1 slope (TS). In the thoracic region, the mean errors for thoracic kyphosis were 5.53°. For the lumbosacral parameters, the mean errors were 1.87° for pelvic tilt (PT) and 5.69° for the lumbar lordosis angle.

### 3.5. Predicted Spinal Parameters of the External Validation Dataset

A comparative analysis was performed with four external validation datasets (Table 5). There were no statistical differences between datasets in all parameters, suggesting that the performance of the artificial intelligence model created was excellent.

## 4. Discussion

Adult spinal deformity (ASD) affects a significant proportion of the elderly population, with 32–68% of individuals over 65 experiencing this condition [12,13,14]. The causes of ASD are diverse, including conditions such as de novo scoliosis, progressive adolescent idiopathic scoliosis, degenerative hyperkyphosis, and iatrogenic flat back deformity [15]. A comprehensive radiographic assessment of the entire spine, including the hip joints, is crucial for evaluating sagittal balance in ASD. Various studies have established the relationship between key spinopelvic parameters and health-related quality of life outcomes, as well as the success of ASD corrective surgeries [16]. These parameters, both regional and global, are vital for disease classification and preoperative planning, offering insights into the overall sagittal balance by considering factors such as cervical hyperlordosis, thoracic hypokyphosis, and pelvic retroversion, independent of postural changes and body size differences [17]. However, manually measuring these parameters can be time-consuming and subject to interobserver variability. Our study introduced a DL model that shows performance comparable to that of human observers in accurately measuring 15 critical sagittal spinal parameters across various spinal conditions.

Numerous studies have applied DL techniques to analyze plain radiographs of the lateral spine automatically. For instance, in a study conducted by Weng et al. [18], a DL model based on an advanced ResUNet architecture was developed for the automatic measurement of the sagittal vertical axis (SVA), demonstrating exceptional reliability compared with human expert assessments. The scope of automatic measurements in whole-spine lateral radiographs has been broadened to include various spinopelvic parameters, such as pelvic incidence, sacral slope, and PT. These measurements have shown not only acceptable error margins but also robust correlations with ground truth values [19]. Further, a study by Yeh et al. [20] reported that the automatic predictions of spinopelvic parameters utilizing a sophisticated two-stage DL model were on par with the reliability of human experts, even in cases involving complex spinal disorders. This underscores the increasing efficacy and reliability of DL applications in spinal radiographic analyses. Galbusera et al. attempted to calculate the spine angles automatically using standardized biplanar images from the EOS system [19]. Despite standardization, this approach also demonstrated the potential for improvement in angle calculation. Other initiatives have focused on 3D spinal reconstruction using both automatic and semiautomatic models. One such study applied a statistical model and a convolutional neural network to reconstruct the shape of the spine precisely, assessing the model accuracy through the Euclidean distance between predictions and actual measurements. Manual intervention was required before the relevant parameters could be calculated.

A key benefit of DL in medical imaging is its ability to provide rapid, objective, and consistent interpretations. Despite advancements in Picture Archiving and Communication Systems (PACS) and specialized commercial software, such as Surgimap (Nemaris, MA, USA), manual identification of points still requires significant professional input and considerable time. While a few studies have reported automatic curvature feature analyses in various spinal imaging modalities [21], these have not been widespread. A notable advancement in this area is the use of annotated vertebral centers for spline-based curve angle measurements. As demonstrated in a recent study [22], this approach yields higher intrarater and interrater reliability than traditional manual Cobb angle measurements, especially in anteroposterior spinal radiographs. However, it is important to note that much of this research has predominantly concentrated on analyzing the frontal plane curvature, with less emphasis on the sagittal plane, highlighting a potential area for development in spinal imaging analysis.

Weng et al. created an artificial intelligence model that analyzed the curvature of the entire spine by detecting the inflection points and apices [23]. Point detection in spinal sagittal curvatures has been the subject of extensive research in both healthy and pathological contexts [24]. Biomechanically, inflection points signify transitional areas between different sagittal curves, while apices influence the distribution of lumbar lordosis [25]. Therefore, achieving accurate relocation of the inflection points and apices and restoring the ideal sagittal profile are critical for spinal surgical procedures. However, because it does not find points to accurately measure parameters, it has the limitation of estimating parameters using a virtual curvature line through inflection points and apices. In this study, we increased the efficiency of angle measurements by directly detecting the points required for angle measurement using artificial intelligence. Although our DL model significantly reduces manual labeling efforts, incorporating a human review process into real clinical settings is advisable.

This study had some limitations. First, although radiological examinations from a multicenter study were used for external validation, the overall dataset size was small. Second, images with atypical vertebral counts were excluded, implying that the model may not accurately predict cases with anomalies such as lumbosacral transitional vertebrae. Third, the predictions were based solely on lateral radiographs, whereas a biplanar EOS system with 3D reconstruction might offer more comprehensive assessments of spinal deformities. Fourth, the performance of the DL model may vary across different spinal conditions as radiographs include a wide range of spinal issues. Despite these limitations, our DL model demonstrated the ability to interpret sagittal spinal curves automatically and consistently.

## 5. Conclusions

The landmark localizer showed the highest accuracy in identifying cervical landmarks, with a median error of 1.5–2.4 mm. External validation was performed using data from four other institutions and good results were obtained. The proposed automatic alignment analysis system identified the positions of the anatomical landmarks of the spine with high precision and generated various radiograph imaging parameters that had a good correlation with manual measurements.

## Figures and Tables

**Table 1:** Names and descriptions of landmarks annotated on whole-spine lateral X-ray.

Name	Description
FH_1	Center of the Femur head
FH_2	Center of the Femur head
S_1	Anterior point of the upper endplate of the sacrum
S_2	Posterior point of the upper endplate of the sacrum
L1_1	Anterior point of the upper endplate of the L1 vertebra
L1_2	Posterior point of the upper endplate of the L1 vertebra
L4_1	Anterior point of the upper endplate of the L4 vertebra
L4_2	Posterior point of the upper endplate of the L4 vertebra
T4_1	Anterior point of the upper endplate of the T4 vertebra
T4_2	Posterior point of the upper endplate of the T4 vertebra
T12_1	Anterior point of the lower endplate of the T12 vertebra
T12_2	Posterior point of the lower endplate of the T12 vertebra
T1	Center of the T1 vertebral body
Forehead	Forehead
FM_1	Anterior point of the foramen magnum
FM_2	Posterior point of the foramen magnum
ODT	Odontoid
Jaw	Jaw
C2_1	Anterior point of the lower endplate of the C2 vertebra
C2_2	Posterior point of the lower endplate of the C2 vertebra
C7	Center of the C7 vertebral body
C7_1	Anterior point of the lower endplate of the C7 vertebra
C7_2	Posterior point of the lower endplate of the C7 vertebra
C7_3	Posterior point of the upper endplate of the C7 vertebra
T1_1	Anterior point of the upper endplate of the T1 vertebra
T1_2	Posterior point of the upper endplate of the T1 vertebra

**Table 2:** Names and measurement methods of spinal parameters measured from landmarks detected in whole-spine lateral X-ray.

Name		Measurement
PI	Pelvic Incidence	The angle between the line connecting the center of femur heads and the center of the sacrum’s upper endplate, and the perpendicular line of the sacrum’s upper endplate.
PT	Pelvic Tilt	The angle between the line connecting the center of the femur heads and the center of the sacrum’s upper endplate, and the vertical.
SS	Sacral Slope	The angle between the sacrum’s upper endplate and the horizontal.
LL	Lumbar Lordosis	The angle between the upper endplate of L1 and the endplate of the sacrum.
L4S1	L4S1 Lordosis	The angle between the upper endplate of L4 and the endplate of the sacrum.
TK	Thoracic Kyphosis	The angle between the upper endplate of T4 and the lower endplate of T12.
TPA	T1pelvic Angle	The angle between the line connecting the center of the T1 vertebral body and the center of the femur heads, and the line connecting the center of the femur heads and the center of the sacrum’s upper endplate.
CBVA	Chin-Brow Vertical Angle	The angle between the line connecting the forehead and chin, and the vertical.
C2C7	C2C7 Angle (Cervical Lordosis Angle)	The angle between the lower endplate of C2 and the lower endplate of C7.
TS	T1 Slope	The angle between the upper endplate of T1 and the horizontal.
TS-CL	T1 Slope—Cervical Lordosis	T1 slope minus cervical lordosis.
ODHA	Odontoid hip axis angle	The angle between the line connecting the odontoid to the center of femur heads, and the vertical.
PI-LL	Pelvic Incidence—Lumbar Lordosis	Pelvic Incidence minus Lumbar Lordosis
SSA	Spino-Sacral Angle	The angle between the line connecting the center of the C7 body and the center of the sacrum’s upper endplate, and sacrum’s upper endplate.
SVA	Sagittal Vertical Axis	Distance between the vertical line at the center of the C7 body and a posterior point of the sacrum’s upper endplate.

**Table 3:** Inter-rater reliability between the two human experts and developed deep learning model.

Parameters	R1 versus R2	DL versus R1	DL versus R2
PI (°)	0.978	0.891	0.889
PT (°)	0.981	0.923	0.915
SS (°)	0.962	0.905	0.897
LL (°)	0.957	0.921	0.915
L4S1 (°)	0.961	0.901	0.894
TK (°)	0.979	0.945	0.931
TPA (°)	0.945	0.894	0.884
CBVA (°)	0.951	0.907	0.901
C2C7 (°)	0.947	0.887	0.881
TS (°)	0.923	0.915	0.909
TS-CL (°)	0.914	0.909	0.897
ODHA (°)	0.928	0.903	0.891
PI-LL (°)	0.927	0.896	0.884
SSA (°)	0.944	0.945	0.925
SVA (mm)	0.957	0.912	0.902

PI, pelvic incidence; PT, pelvic tilt; SS, sacral slope; LL, lumbar lordosis; L4S1, L4S1 lordosis; TK, thoracic kyphosis; TPA, T1 pelvic angle; CBVA, chin-brow vertical angle; C2C7, C2C7 angle; TS, T1 slope; TS-CL, T1 slope—cervical lordosis; ODHA, odontoid hip axis angle; PI-LL, pelvic incidence—lumbar lordosis; SSA, spino-sacral angle; SVA, sagittal vertical axis.

**Table 4:** Performance evaluation of the spinal parameters of the deep learning model.

Parameters	Ground Truth	Parameter Error	Correlation Analysis		Wilcoxon Signed-Rank Test
			R	*p* Value	*p* Value
PI (°)	53.8 ± 18.8°	2.6 ± 3.1°	0.982	<0.001 *	0.497
PT (°)	14.8 ± 11.3°	1.8 ± 2.2°	0.917		0.512
SS (°)	39.4 ± 7.9°	2.2 ± 3.4°	0.912		0.459
LL (°)	41.2 ± 17.3°	5.7 ± 3.5°	0.991		0.279
L4S1 (°)	30.7 ± 11.6°	4.5 ± 2.8°	0.857		0.247
TK (°)	27.2 ± 11.2°	5.5 ± 4.5°	0.812		0.078
TPA (°)	24.9 ± 23.2°	1.8 ± 1.1°	0.792		0.758
CBVA (°)	1.8 ± 5.2°	0.7 ± 0.6°	0.984		0.678
C2C7 (°)	13.6 ± 9.7°	5.5 ± 6.5°	0.845		0.598
TS (°)	22.8 ± 10.2°	5.7 ± 6.2°	0.784		0.084
TS-CL (°)	9.8 ± 2.4°	4.1 ± 5.9°	0.809		0.097
ODHA (°)	4.3 ± 5.4°	0.2 ± 0.2°	0.978		0.594
PI-LL (°)	12.1 ± 7.5°	3.0 ± 4.5°	0.962		0.596
SSA (°)	120.1 ± 12.4°	3.3 ± 2.5°	0.927		0.492
SVA (mm)	22.1 ± 19.2 mm	3.0 ± 2.9 mm	0.986		0.745

PI, pelvic incidence; PT, pelvic tilt; SS, sacral slope; LL, lumbar lordosis; L4S1, L4S1 lordosis; TK, thoracic kyphosis; TPA, T1 pelvic angle; CBVA, chin-brow vertical angle; C2C7, C2C7 angle; TS, T1 slope; TS-CL, T1 slope—cervical lordosis; ODHA, odontoid hip axis angle; PI-LL, pelvic incidence—lumbar lordosis; SSA, spino-sacral angle; SVA, sagittal vertical axis; * *p* value < 0.05.

**Table 5:** Predicted spinal parameters of the external validation dataset.

Parameters	Ground Truth	Parameter Error	External-Validation Dataset 1 Error	External-Validation Dataset 2 Error	External-Validation Dataset 3 Error	External-Validation Dataset 4 Error	*p*-Value
PI (°)	53.8 ± 18.8°	2.7 ± 3.1°	3.3 ± 2.1°	2.2 ± 3.9°	4.2 ± 2.4°	3.6 ± 2.1°	0.479
PT (°)	14.8 ± 11.3°	1.9 ± 2.2°	2.7 ± 2.0°	2.2 ± 2.7°	2.5 ± 1.2°	2.3 ± 1.3°	0.545
SS (°)	39.4 ± 7.9°	2.2 ± 3.4°	2.3 ± 3.3°	3.6 ± 2.2°	3.8 ± 2.4°	3.6 ± 3.0°	0.471
LL (°)	41.2 ± 17.3°	5.7 ± 3.5°	5.1 ± 3.0°	6.2 ± 4.4°	5.6 ± 3.6°	4.2 ± 3.3°	0.784
L4S1 (°)	30.7 ± 11.6°	4.5 ± 2.8°	5.2 ± 2.5°	4.2 ± 2.6°	4.4 ± 3.1°	5.0 ± 2.4°	0.612
TK (°)	27.2 ± 11.2°	5.5 ± 4.5°	5.9 ± 4.4°	5.9 ± 5.2°	4.2 ± 3.8°	5.0 ± 4.4°	0.274
TPA (°)	24.9 ± 23.2°	1.8 ± 1.1°	1.4 ± 1.8°	1.9 ± 1.7°	1.9 ± 1.9°	1.5 ± 1.1°	0.798
CBVA (°)	1.8 ± 5.2°	0.7 ± 0.6°	0.6 ± 0.4°	0.4 ± 0.2°	0.8 ± 1.4°	0.8 ± 1.0°	0.571
C2C7 (°)	13.6 ± 9.7°	5.5 ± 6.5°	4.6 ± 4.4°	5.4 ± 5.2°	4.8 ± 5.4°	5.8 ± 4.0°	0.435
TS (°)	22.8 ± 10.2°	5.7 ± 6.2°	4.4 ± 4.4°	5.1 ± 6.1°	5.7 ± 4.6°	5.4 ± 6.4°	0.645
TS-CL (°)	9.8 ± 2.4°	4.1 ± 5.9°	4.5 ± 6.3°	4.1 ± 5.3°	3.9 ± 4.4°	3.7 ± 4.8°	0.421
ODHA (°)	4.3 ± 5.4°	0.2 ± 0.2°	0.1 ± 0.4°	0.1 ± 0.2°	0.1 ± 0.3°	0.3 ± 0.9°	0.764
PI-LL (°)	12.1 ± 7.5°	3.0 ± 4.5°	3.1 ± 4.9°	2.0 ± 2.7°	2.4 ± 4.8°	2.1 ± 3.2°	0.841
SSA (°)	120.1 ± 12.4°	3.3 ± 2.5°	3.2 ± 2.6°	4.0 ± 2.48°	3.1 ± 2.4°	3.9 ± 2.5°	0.623
SVA (mm)	22.1 ± 19.2 mm	3.0 ± 2.9 mm	2.0 ± 2.5 mm	2.9 ± 2.5 mm	2.7 ± 1.1 mm	2.9 ± 1.5 mm	0.812

PI, pelvic incidence; PT, pelvic tilt; SS, sacral slope; LL, lumbar lordosis; L4S1, L4S1 lordosis; TK, thoracic kyphosis; TPA, T1 pelvic angle; CBVA, chin-brow vertical angle; C2C7, C2C7 angle; TS, T1 slope; TS-CL, T1 slope—cervical lordosis; ODHA, odontoid hip axis angle; PI-LL, pelvic incidence—lumbar lordosis; SSA, spino-sacral angle; SVA, sagittal vertical axis.

## Data Availability

Data are contained within the article.
